# Traveling into the Abyss: Risk Perception of German Travelers at the Onset of the COVID-19 Pandemic

**DOI:** 10.4269/ajtmh.20-0892

**Published:** 2020-08-18

**Authors:** Parichehr Shamsrizi, Johannes Jochum, Benno Kreuels, Michael Ramharter

**Affiliations:** Department of Tropical Medicine, Bernhard Nocht Institute for Tropical Medicine & I Department of Medicine, University Medical Center Hamburg-Eppendorf, Hamburg, Germany

## Abstract

The emergence and international spread of SARS-CoV-2 led to unprecedented challenges for international travelers including health-related concerns and international travel restrictions. Remarkably, overseas travelers consulted at our travel clinic during the first quarter of 2020 were apparently not disconcerted by the evolving pandemic with a continuously high rate of consultations at our center; 85% of travelers did not actively inquire about COVID-19 during the pretravel consultation including individuals with clinically significant immunosuppression constituting a high-risk group for COVID-19–related adverse health outcome. This experience demonstrates the societal responsibility of travel medicine practitioners to proactively provide unbiased information about the health-related and travel-related impact of newly emerging infections.

COVID-19 is a prime example of a rapidly spreading emerging infection with international travel as a main route of geographical spread.^1^ The dynamic spread of SARS-CoV-2 virus was followed by an unparalleled political response in affected countries, leading to unprecedented travel restrictions and at times to a near to complete halt of international air travel. Travelers were suddenly confronted with a dynamic epidemiological situation of an emerging viral infection, largely unknown risks for personal health during travel, and unpredictable travel restrictions with the potential to impede return to home countries. This situation led to an exceptional challenge for travel medicine practitioners to quickly and adequately respond in their daily practice to the evolving situation. To allow for informed and adequate travel recommendations, an understanding of the risk perception of travelers with regard to the evolving COVID-19 pandemic is of importance. Here, we evaluated how travelers perceived this risk at the onset of the international outbreak.

Our travel clinic at the University Medical Center Hamburg is one of the largest travel clinics in Germany providing pretravel advice and travel-related vaccinations as well as health care for returning travelers. Despite reports of an international spread of COVID-19 in January 2020, the number of pretravel consultations at our center remained constant at 200–300 consultations per week (Figure 1). Similarly, the dramatic increase in the number of global cases of COVID-10 in February and first cases in Germany in week 5 had no impact on interest in overseas travel by our clients. To better understand this apparent discrepancy, we collected information about the perception of COVID-19 by clients planning imminent overseas travel between February 27, 2020 and the March 6, 2020 at the onset of the outbreak in Europe as part of an institutional quality assurance program.

**Figure 1.**
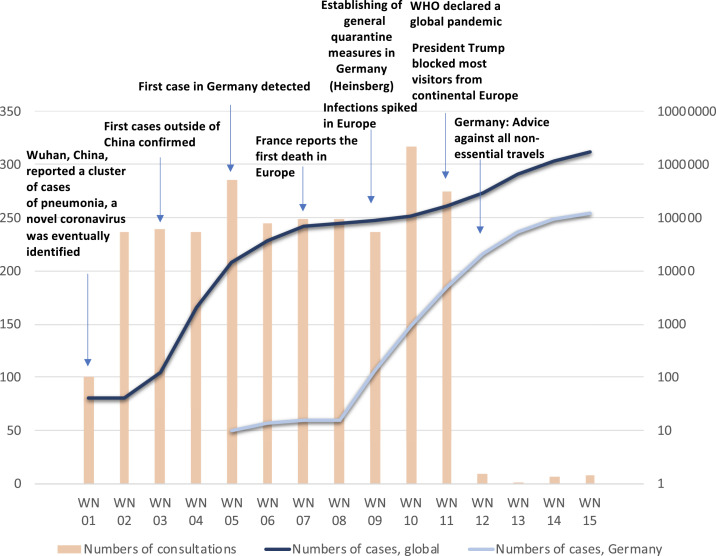
Number of cases (Germany and global) and number of consultations at the onset of the COVID-19 pandemic. This figure appears in color at www.ajtmh.org.

Overall, 85% of the clients (218 of 258) did not inquire about information about the COVID-19 outbreak during the consultation; 21% of those planned trips to East and Southeast Asia—the regions most affected by COVID-19 at that time. The most frequent other travel destinations were Africa and Latin America, regions for which only scarce epidemiological information was available at that time because of limited testing capacity in these regions. And, 40 of 258 (16%) travelers actively inquired about the current COVID-19 situation and how this might affect their travel; 11 of the 40 (28%) had serious concerns about their trip and asked for recommendations whether they should cancel the trip under these circumstances. The remaining 29 of 40 (73%) clients mentioned the outbreak with minor concern, among whom 11 were flying to Southeast Asia. Three patients with clinically significant immunosuppression and, therefore, considered to be among the highest risk groups for adverse health outcome did not inquire about COVID-19.

In the same period of time, the number of confirmed cases started to increase exponentially in Germany, as shown in Figure 1, and case numbers in many travel destinations increased concomitantly.^2^ Five days after the end of our assessment, a global pandemic was declared by the Secretary General of the United Nations, and another 5 days later, the German Federal Foreign Office advised against all nonessential travel to other countries, and flight connections were stopped to most foreign countries.

Despite the dramatic epidemiological situation and the importance of international travel as route of intercontinental transmission, the vast majority of travelers at our center did not appreciate the health risks and logistical challenges posed by the evolving pandemic just before the international ban on travel and the near to complete lockdown on international air travel. This was in stark contrast to the growing concern in Germany at the same time.^3^ Already since the end of January, panic buying and hoardings were reported from German communities. To our understanding, this experience astoundingly demonstrates that the societal response to an imminent epidemiological threat by a viral emerging infection is not uniform in a population such as in Germany with highly concerned people on one end of the spectrum and individuals negating any risk on the other end. Despite the many limitations of this survey including small sample size, limited information about participants, and reasons for travel, this experience highlights the responsibility and societal duty of travel medicine specialists to proactively address and to actively provide balanced information about health risks and travel challenges associated with major pandemics such as COVID-19.
